# A multicenter, prospective, randomized, controlled trial evaluating the safety and efficacy of intracoronary cell infusion mobilized with granulocyte colony-stimulating factor and darbepoetin after acute myocardial infarction: study design and rationale of the 'MAGIC cell-5-combination cytokine trial'

**DOI:** 10.1186/1745-6215-12-33

**Published:** 2011-02-07

**Authors:** Hyun-Jae Kang, Min-Kyung Kim, Myung-Gon Kim, Dong-Ju Choi, Jung-Han Yoon, Young-Bae Park, Hyo-Soo Kim

**Affiliations:** 1 Division of Cardiology, Department of Internal Medicine, Seoul National University Hospital, 28 Yongon-dong, Jongno-gu, Seoul, Korea; 2 Healthcare System Gangnam Center, Seoul National University Hospital, 737 Yeoksam-dong, Gangnam-gu, Seoul, Korea; 3 Division of Cardiology, Department of Internal Medicine, Kyunghee University Medical Center, 1 Hoegi-dong, Dongdaemun-gu, Seoul, Korea; 4 Cardiovascular Center, Seoul National University Bundang Hospital, 166 Gumiro, Bundang-gu, Seongnam, Korea; 5 Division of Cardiology, Department of Internal Medicine, Wonju Christian Hospital, 162 Ilsan-dong, Wonju, Korea

## Abstract

**Background:**

Bone marrow derived stem/progenitor cell transplantation after acute myocardial infarction is safe and effective for improving left ventricular systolic function. However, the improvement of left ventricular systolic function is limited. This study will evaluate novel stem/progenitor cell therapy with combination cytokine treatment of the long-acting erythropoietin analogue, darbepoetin, and granulocyte colony-stimulating factor (G-CSF) in patients with acute myocardial infarction.

**Methods:**

The 'MAGIC Cell-5-Combination Cytokine Trial' is a multicenter, prospective, randomized, 3-arm, controlled trial with blind evaluation of the endpoints. A total of 116 patients will randomly receive one of the following three treatments: an intravenous darbepoetin infusion and intracoronary infusion of peripheral blood stem cells mobilized with G-CSF (n = 58), an intracoronary infusion of peripheral blood stem cells mobilized with G-CSF alone (n = 29), or conventional therapy (n = 29) at phase I. Patients with left ventricular ejection fraction < 45% at 6 months, in the patients who received stem cell therapy at phase I, will receive repeated cell therapy at phase II. The objectives of this study are to evaluate the safety and efficacy of combination cytokine therapy with erythropoietin and G-CSF (phase I) and repeated progenitor/stem cell treatment (phase II).

**Discussion:**

This is the first study to evaluate the safety and efficacy of combination cytokine based progenitor/stem cell treatment.

**Trial registration:**

http://www.ClinicalTrials.gov identifier: NCT00501917.

## Backgrounds

Recent clinical trials reported favorable effects of stem/progenitor cell transplantation in patients with acute myocardial infarction (AMI), suggesting that stem cell transplantation is feasible, safe, and effective for improvement of left ventricular (LV) systolic function and myocardial perfusion [1-3]. Granulocyte colony-stimulating factor (G-CSF) alone is mostly ineffective for improvement of LV systolic function [4]. But G-CSF is effective for stem/progenitor cell mobilization and local delivery of mobilized stem/progenitor cell by G-CSF improved cardiac function in patients with myocardial infarction [2,3,5-8]. However, degree of improvement by G-CSF based stem/progenitor cell therapy is modest and similar to that of bone marrow stem cell therapy. There are reasonable explanations of limited efficacy of current stem/progenitor cell therapy using G-CSF, such as, low homing-efficiency, poor long-term survival of infused stem/progenitor cells, and potential dysfunction of mobilized stem/progenitor cells by G-CSF [9,10].

To overcome potential limitations of current stem/progenitor cell therapy and to improve efficacy, combination treatment with multiple cytokines is introduced in this study. Erythropoietin is a cytokine secreted by the kidney in response to hypoxia, and regulates plasma hemoglobin concentrations. Experimental studies revealed that erythropoietin can protect cardiomyocytes from necrotic or apoptotic damage by ischemia [11]. Moreover, it can induce angiogenesis by stimulating endothelial progenitor cells (EPCs) [12,13]. In preclinical study, combination therapy with erythropoietin and bone marrow stem cells showed better outcomes than monotherapy either with bone marrow stem cell or erythropoietin [14]. Expecting additional effects by erythropoietin, we have developed a novel stem/progenitor cell treatment strategy for AMI, combining erythropoietin to stem/progenitor cell therapy using G-CSF. Additionally we planned to evaluate safety and efficacy of repeated stem cell therapy in severely diseased patients who have persistent LV dysfunction at 6 months after initial stem/progenitor cell therapy. Here, we describe our new treatment strategy for the first time and present the rationale of this study by reviewing the current evidence for a beneficial effect of G-CSF-based stem/progenitor cell therapy and erythropoietin in patients with AMI.

## Methods

### Study Objectives

The objectives of the present study are (1) to evaluate whether novel combination stem/progenitor cell therapy can improve LV systolic function better than conventional therapy and intracoronary infusion of mobilized peripheral blood stem/progenitor cell (PBSC) by G-CSF alone respectively; and (2) to evaluate the safety and feasibility of combination use of G-CSF and darbepoetin.

The primary end point is the changes in resting LV systolic function (ejection fraction) in patients after AMI, measured by cardiac magnetic resonance imaging (MRI) 6 months after primary revascularization of culprit arteries and stem/progenitor cell transplantation.

The secondary end points of the study are change of wall motion score index measured by cardiac cine-MRI or echocardiography, and the major adverse cardiac events (MACE), defined as all cause mortality, target lesion revascularization, rehospitalization for recurrent ischemia or heart failure, or refractory angina. And in case of repeated stem/progenitor cell therapy, changes of LV ejection fraction 6 months after repeated therapy.

### Study design

**Figure 1:**
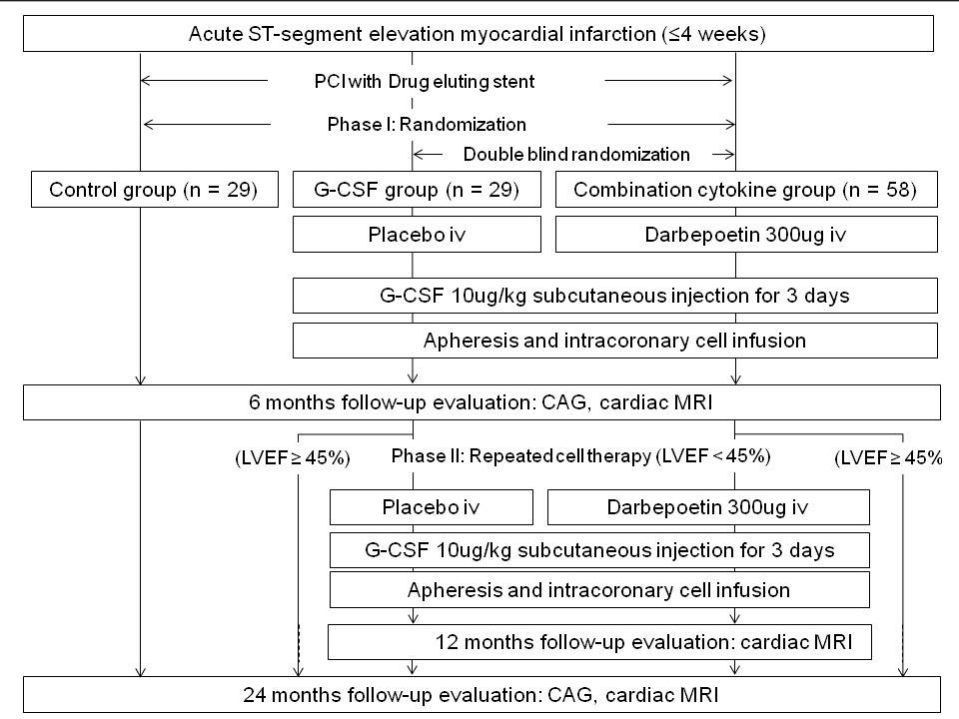
**Flow chart demonstrating scheduling during the MAGIC Cell-5-Combination Cytokine Trial**.

The 'MAGIC Cell-5-Combination Cytokine Trial' described here is a multicenter, prospective, randomized, 3-arm, controlled, phase 2 trial with blind evaluation of endpoints (Figure 1).

**Phase I **After a successful revascularization of culprit lesion with drug eluting stents, patients will be randomly allocated and receive one of three treatments: (1) for the combicytokine group (n = 58) intravenous infusion of long acting analogue of erythropoietin, darbepoetin (Amgen, USA) just after revascularization and an additional intracoronary infusion of PBSC mobilized with G-CSF, (2) for the G-CSF group (n = 29) an intracoronary infusion of PBSC mobilized with G-CSF alone, or (3) for the control group (n = 29) conventional therapy. Patients will undergo cardiac MRI within a week after revascularization.

**Phase II **For the G-CSF and combicytokine groups, patients who still have LV systolic dysfunction (LV ejection fraction < 45%) at 6 months after initial stem/progenitor cell therapy, will receive repeated cell therapy as they are assigned in phase I.

### Study population

In total, 116 patients with acute ST segment elevation acute myocardial infarction (STEMI) will be included. Patients will be considered for participation in this study if they meet all of the inclusion criteria and none of the exclusion criteria in Table 1. After receiving the angiographies, patients and/or their family will be asked for their oral informed consent by an independent coordinator; if they agree, patients will be randomly allocated by means of sealed envelope to one of the 3 treatment groups. After being admitted to the coronary care unit and after revascularization, patients will sign the written informed consent. For the phase II therapy, the patients who meet the inclusion criteria and their LV ejection fractions under 45% at 6 months will be eligible for the repeated cell therapy.

**Table 1:** Enrollment criteria for the MAGIC Cell-5-Combination Cytokine Trial.

Inclusion criteria
Age: 18 to 80 years
Able to verbally confirm understandings of risks, benefits, and treatment alternatives of receiving the cell therapy provides written informed consent before any study-related procedure
ST-segment elevation acute myocardial infarction
Successful revascularization of culprit vessel

Exclusion criteria

Known hypersensitivity or contraindication to any of the following medications:
Heparin, aspirin, clopidogrel, sirolimus, everolimus, contrast media
Uncontrolled congestive heart failure (patients with LVEF <20% or Killip class II, III or those with cardiogenic shock)
Uncontrolled myocardial ischemia (repeated chest pain or dyspnea after revascularization)
Uncontrolled ventricular arrhythmia
History of malignancy
Serious hematologic disease
Current infectious disease needs antibiotics therapy
Creatinine level >2.0 mg/dL or dependence on dialysis

LVEF, left ventricular ejection fraction

### Treatment

#### Phase I

All patients will be treated, according to current practice guidelines and standard medication for an AMI consisting of aspirin, clopidogrel, heparin and possibly abciximab will be administered during percutaneous coronary intervention [15]. Patients randomized to the combicytokine group will receive 4.5 ug/kg of intravenous darbepoetin (maximum: 300 ug) infusion just after revascularization. Patients in the G-CSF group will receive placebo infusion. Infusion of darbepoetin or placebo will be performed by double blind manners. Patients in the control group will not receive placebo and treated in un-blinded state.

After revascularization, patients in the combicytokine and G-CSF group will also be treated with an intracoronary infusion of PBSC. Patients will receive a subcutaneous injection of G-CSF (Dong-A Pharmaceutical, South Korea) at 5 ug/kg body weight twice daily for 3 days. After 3 days of G-CSF injection, mobilized PBSC will be collected with a COBE spectra apheresis system (COBE BCT Inc., USA) using the mononuclear cell collection method. The infusion cell doses will be 2 × 10^9 ^monocytes per patient, in order to guarantee the minimum target cell dose of 7 × 10^6 ^CD34-positive cells. Collected autologous PBSC will be selectively infused to infarct-related artery via over-the-wire balloon catheter, as previously described^2^. Placebo will not be applied to the control group.

#### Phase II

Open follow-up will be made after 6 months follow-up evaluation. Phase II trial is considered for the patients in the G-CSF and combicytokine group, whose LV ejection fraction is under 45% at 6 months evaluated by cardiac MRI. Eligible patients will be asked to receive repeated stem/progenitor cell therapy as they were assigned in phase I. If the patients agree with the repeated therapy, darbepoetin or placebo injection will be given and then stem cell mobilization followed by intracoronary infusion will be performed under the same way, as described in the previous paragraph.

### Cardiac MRI

Cardiac contrast enhanced MRI (Sonata 1.5T, Siemens, Germany) will be performed after revascularization within a week before PBSC infusion. After localization of the heart, 8 to 11 contiguous short-axis slices of 8 mm thickness with gap of 2 mm will be acquired to cover entire left ventricle from base to apex. After intravenous application of gadolinium-diethylene-triamine penta-acetate (GE healthcare, USA), late enhancement imaging will be performed with a phase-sensitive inversion recovery sequence. LV ejection fraction and LV volumes will be calculated with ARGUS software (Siemens). Regional LV function will be assessed by determining systolic wall motion with a 17-segment model, as proposed by the American Heart Association [16]. Segmental wall thickening will be judged visually as falling into 1 of the following semiquantitative categories: normokinetic (1), hypokinetic (2), akinetic (3) or dyskinetic (4). Segmental extent of late enhancement will be scored as one of the following: 0%, 1 to 25%, 26 to 50%, 51 to 75%, 76 to 100% of either volume extent or transmural extent.

### Patient follow-up

Follow-up cardiac MRIs and coronary angiographies for all patients will be performed 6, and 24 months after AMI (Figure 1). Additional cardiac MRI evaluation will be performed 6 months after repeated stem cell therapy. Clinical follow-ups (including patient interviews, physical assessments, and clinical laboratory tests) will be conducted every 2-4 months throughout the study period. During follow up all patients will be treated, according to current practice guidelines, with antiplatelet agents, a statin, a beta-blocker, and an angiotensin converting enzyme inhibitor or angiotensin-receptor blocker, unless these agents are contraindicated [17]. If patients who were enrolled in this study but did not complete stem cell infusion drop out, we will follow them at least till 1 month after index hospitalization.

### Sample size calculation and statistical analysis

This study was designed to detect differences in changes of LV ejection fraction at 6 months relative to baseline between the combicytokine group and two other groups respectively. Sample size was calculated on the premise that we would assign patients to the combicytokine groups and two comparator group (the control and G-CSF group) at a ratio of 2:1 respectively to adequately evaluate the effects of new treatment with combicytokine. We hypothesized that combination therapy with darbepoetin can induce 2.4% additional improvement of LV ejection fraction than the G-CSF group, which was 60% improvement over reference value observed in small pilot study and our previous study [3,18]. This study has 80% power to detect a 5% difference between treatment groups, assuming a 1-sided alpha of 0.05. With an estimated drop-out rate of 10%, we will need approximately 29 patients in the control and G-CSF group respectively and 58 patients in the combicytokine group.

The primary and secondary endpoints will be analyzed on modified intention-to-treat analysis. For evaluation of primary and secondary efficacy endpoints, patients who completed stem cell infusion will be included. For evaluation of safety endpoints, all patients who signed the written informed consent and were randomized to receive any part of active treatment (darbepoetin/placebo infusion and G-CSF, PBSC infusion) will be included in the analysis.

Baseline characteristics of study patients will be summarized in terms of frequencies and percentages for categorical variables and by means with standard deviations for continuous variables. Categorical variables will be compared with Chi-square or Fisher's exact tests. Continuous variables showing normal distribution will be compared using the student t-test whereas those showing non-normal distribution will be compared using the nonparametric Wilcoxon 2-sample test between the combi-cytokine group and two comparators, the G-CSF group and the control group, respectively. Bonferroni's correction will be applied to the repeated comparisons. Significance will be defined as p value < 0.05.

### Trial Organization

#### Executive committee

The executive committee will be composed of the study chairperson and the principal investigators of the investigating centers. This committee is responsible for overseeing the administrative progress of the study and will approve the final trial design and protocol issued to the data and safety monitoring board (DSMB) and the clinical sites. This committee will also be responsible for reviewing the final results, determining the methods of presentation and publication, and selecting secondary projects and publications by members of the steering committee. The executive committee also holds the right to modify or stop the study prematurely based on recommendations from the DSMB.

#### Steering committee

The steering committee will be composed of the principal investigators from the centers participating in this trial. The committee is responsible for the day-to-day administrative management of the trial and will meet on a regular basis to monitor subject enrollment, clinical site progress, and protocol compliance. It will be the responsibility of the steering committee to provide assistance and education to individual sites and researchers for trial management, record keeping, and reporting requirements. The steering committee will prepare reports to be reviewed by the executive committee.

#### Data safety monitoring board

The DSMB will be composed of general and interventional cardiologists, and a biostatistician. The DSMB will function in accordance with applicable regulatory guidelines. The board members are independent and will not be participating in the trial. The DSMB committee will review the safety data from this study and make recommendations based on safety analyses of serious adverse events, protocol deviations, and 30-day follow-up reports. The frequency of the DSMB meetings will be determined prior to commencement of the study. Additionally, the DSMB may call a meeting at any time if there is reason to suspect that safety is an issue. The DSMB is responsible for making recommendations regarding any safety or compliance issues throughout the course of the study and may recommend that the executive committee modify or stop the study. However, all final decisions regarding study modifications rest with the executive committee. All cumulative safety data will be reported to the DSMB and reviewed on an ongoing basis throughout enrollment and follow-up periods to ensure patient safety. Every effort will be made to allow the DSMB to conduct an unbiased review of patient safety information. Before the DSMB's first review of the data, the DSMB charter will be drafted. The DSMB will develop a consensus understanding of all trial end points and definitions used in the event adjudication process. All DSMB reports will remain strictly confidential but will be made available to the regulatory body upon request.

#### Clinical event adjudication committee

The clinical event adjudication committee (CEAC) is composed of interventional and noninterventional cardiologists who are not participants in the study. The CEAC is charged with the development of specific criteria used for the categorization of clinical events and clinical endpoints in the study, based on study protocols. At the onset of the trial, the CEAC will establish explicit rules outlining the minimum amount of data required and the algorithm to follow when classifying a clinical event. All members of the CEAC will be blinded to the primary results of the trial. The CEAC will meet regularly to review and adjudicate all clinical events in which the required minimum data are available. The committee will also review and rule on all deaths that occur throughout the trial.

#### Data coordination and site management

Data coordination and site management services will be performed by dedicated research nurses from the clinical trials center at Seoul National University Hospital. The designated trial monitors, at appropriate intervals, will review investigational data for accuracy and completeness and ensure compliance with the protocols.

### Ethical approval

This study has been approved by the institutional review board of Seoul National University Hospital.

## Discussion

Erythropoietin has several non-erythropoietic functions. Erythropoietin was found to be a potent stimulus for the mobilization of EPCs into peripheral blood, which was associated with neovascularization of ischemic tissue [18]. A pilot study of 22 patients with acute STEMI evaluated the safety and tolerability of a single-bolus 300 ug darbepoetin treatment administered before primary PCI [19]. No adverse events were noted during the 30-day follow-up. At 72 hours after darbepoetin administration, CD34^+^/CD45^- ^cell count significantly increased in darbepoetin group but not in the control. This result suggests that darbepoetin treatment after myocardial infarction stimulates EPC mobilization.

Erythropoietin and its receptor have also been found in neuronal, vascular, and myocardial tissue [20]. Erythropoietin and its receptor exert antiapoptotic actions, as well as pro-angiogeneic and anti-inflammatory effects [21-23]. Additionally, it increases coronary flow and enhances the synthesis and bioavailability of constitutive nitric oxide, via endothelial nitric oxide synthase transcription and activation [24]. Recently Brunner et al reported that erythropoietin treatment has improved homing of bone marrow cells into the infarcted myocardium, via the CXCR-4/SDF-1-axis [25]. However based on current clinical studies, erythropoietin alone is ineffective to improve cardiac function after AMI.

Suh et al reported that high dose single bolus infusion of erythropoietin failed to reduce infarct size but that it was safe in patients with AMI [26]. Ott et al reported that epoietin-beta infusion just after primary revascularization of STEMI did not improve LV function and infarct size and that there was a trend towards a higher rate of adverse events in the erythropoietin-treated group [27]. Thus, the effort to measure the safety and efficacy of erythropoietin treatment in myocardial infarction patients is still continuing; for instance, the on-going EPAMINONDAS trial is investigating whether erythropoietin treatment can safely reduce infarct size in STEMI patients [28].

Although erythropoietin alone is insufficient to improve cardiac function in  AMI, its cytoprotective and stem cell mobilizing effects can be synergistically integrated to G-CSF based stem/progenitor cell therapy. Effects of G-CSF and erythropoietin are mediated by activation of their own specific receptors. Thus we hypothesized that a combination therapy of G-CSF and darbepoetin will enhance the therapeutic efficacy of stem cell therapy. The 'MAGIC Cell-5-combination cytokine Trial' will allow us evaluate efficacy and safety of new combination strategy. We hope that new strategy proposed here will combine complementary beneficial effects from each of these treatments and will prevent ischemic cardiomyopathy in patients with myocardial infarctions.

Regarding the efficacy of the repeated stem/progenitor cell therapy in myocardial infarction, there have been mixed results [29,30]. With the phase II of this trial, we will evaluate whether repeated PBSC therapy is safe, feasible, and effective for improving cardiac function. In our study, the repeated cell therapy will be given at 6 months after AMI. The optimal timing of repeated stem cell therapy is unknown yet, thus our study may be able to give insight about this issue.

## List of abbreviations used

AMI: acute myocardial infarction; LV: left ventricular; G-CSF: Granulocyte colony-stimulating factor; PBSC: peripheral blood stem/progenitor cell; MACE: major adverse cardiac events; STEMI: ST segment elevation acute myocardial infarction; DSMB: data and safety monitoring board; CEAC: Clinical Event Adjudication Committee;

## Competing interests

The authors declare that they have no competing interests.

## Authors' contributions

All authors had contribution to conception and design of the study, drafting the manuscript and approved submission of the final manuscript.
